# Receptor-Interacting Protein Kinases 1 and 3, and Mixed Lineage Kinase Domain-Like Protein Are Activated by Sublytic Complement and Participate in Complement-Dependent Cytotoxicity

**DOI:** 10.3389/fimmu.2018.00306

**Published:** 2018-02-23

**Authors:** Michal Lusthaus, Niv Mazkereth, Natalie Donin, Zvi Fishelson

**Affiliations:** ^1^Department of Cell and Developmental Biology, Sackler Faculty of Medicine, Tel Aviv University, Tel Aviv, Israel

**Keywords:** complement, C5b-9, receptor-interacting protein kinase 1, receptor-interacting protein kinase 3, mixed lineage kinase domain-like protein, regulated necrosis

## Abstract

The complement system participates in the pathogenesis of many diseases. Complement activation produces several active protein complexes and peptides, including the terminal C5b-9 complexes. It was reported that C5b-9 complexes insert into the plasma membrane and cause membrane perturbation, intracellular calcium surge, metabolic depletion, and osmotic lysis. Previously, we showed that complement-dependent cytotoxicity (CDC) is regulated by JNK and Bid. Here, we demonstrate that three mediators in TNFα-induced necroptosis (regulated necrosis), the receptor-interacting protein kinases, receptor-interacting protein kinase 1 (RIPK1) and receptor-interacting protein kinase 3 (RIPK3), and mixed-lineage kinase domain-like protein (MLKL), are activated by complement and contribute to CDC. Cell treatment with necrostatin-1 (Nec-1), a RIPK1 inhibitor, GSK’872, a RIPK3 inhibitor, or necrosulfonamide and GW806742X, MLKL inhibitors, restrain CDC. These findings were confirmed by using specific siRNAs targeting the synthesis of these proteins. Mouse fibroblasts lacking RIPK3 or MLKL were found to be less sensitive to C5b-9 than were wild-type (WT) fibroblasts. Enhanced CDC was achieved by RIPK1 or RIPK3 overexpression but not by the overexpression of a RHIM-RIPK1 mutant nor by a kinase-dead RIPK3 mutant. Nec-1 reduces the CDC of WT but not of RIPK3-knockout fibroblasts. Cells treated with a sublytic dose of complement exhibit co-localization of RIPK3 with RIPK1 in the cytoplasm and co-localization of RIPK3 and MLKL with C5b-9 at the plasma membrane. Data supporting cooperation among the RIP kinases, MLKL, JNK, and Bid in CDC are presented. These results provide a deeper insight into the cell death process activated by complement and identify potential points of cross talk between complement and other inducers of inflammation and regulated necrosis.

## Introduction

The complement system is a key element of innate and adaptive immunity (1–3). It mediates numerous activities, including the activation of cell death (CD), the opsonization of microorganisms and apoptotic cells, the clearance of immune complex, and the activation of inflammatory as well as anti-inflammatory processes. The classical, lectin, and alternative initiation pathways converge at the stage of terminal complement pathway activation and generate the complement membrane attack complex composed of C5b, C6, C7, C8, and C9, also known as the C5b-9 complex (4). Membrane insertion of C5b-9 triggers dose-dependent life and death signals in target cells. Thus, elevated concentrations of intracellular calcium ions have been implicated both in cell recovery and in CD (5–7). At sublytic doses, C5b-9 can activate pro-survival signals by activating protein kinase C, ERK, Bcl-2, and NF-κB (7–9). At lytic doses, the C5b-9 complexes induce a necrotic-type CD characterized by a leaky plasma membrane. The necrotic CD activated by C5b-9 involves the activation of JNK and Bid (10, 11).

Necroptosis is a regulated necrotic CD type activated by TNFα, FAS-ligand, LPS, interferons, and various other inducers (12–18). Necroptosis has been linked to many pathological conditions and afflictions such as ischemia, neurodegeneration, viral infections, and inflammation (18–21). The receptor-interacting protein kinases 1 and 3 [receptor-interacting protein kinase 1 (RIPK1) and receptor-interacting protein kinase 3 (RIPK3)] are central regulators of this pathway (19, 22, 23). Upon cell stimulation with TNFα, TNFR1 recruits several adaptor proteins to form complex I, which is composed of TRADD, RIPK1, and TRAF2/5 (24, 25). The internalization of ligand-bound TNFR1 leads to the formation of complex II containing deubiquitinated RIPK1, caspase-8, and FADD (26). The latter complex induces apoptosis. However, when caspase-8 is compromised, a necroptosis-inducing complex, namely, a necrosome, is formed with RIPK3 (19, 27–29). The serine/threonine kinase activity of RIPK1 is required for necroptosis (30). Inhibition of the kinase activity of RIPK1 by the allosteric inhibitor necrostatin-1 (Nec-1) prevents RIPK1–RIPK3 interactions and blocks necroptosis (12). RIPK3’s interaction with RIPK1 is an essential step in TNFα-induced programmed necrosis and together they form an oligomeric amyloid-like necrosome through their RIP homotypic interaction (RHIM) motif (27, 29, 31, 32). The kinase-dead mutant of RIPK3, namely, K50A, lacks a RIPK3 pro-necroptotic function in TNFα-treated HT-29 cells (27). RIPK3 mediates TNFα-induced necroptosis by activating mixed lineage kinase domain-like protein (MLKL) (33–37). Their interaction and TNFα-driven necroptosis are allosterically inhibited by necrosulfonamide (NSA) (35).

Here, we determined whether the key activators of necroptosis are activated by complement and are involved in complement-induced necrotic CD. Our results indicate that, at sublytic doses, complement induces RIPK1/RIPK3/MLKL activation and, at lytic doses, it recruits them for necrotic CD. Bid and JNK also appear to be involved in regulating this CD signaling.

## Materials and Methods

### Cells

Human erythroleukemia K562 cells were grown in RPMI-1640 medium (Sigma, Rehovot, Israel) at 37°C, in 5% CO_2_. Human embryonic kidney HEK-293T cells, colon adenocarcinoma HT-29 cells, breast cancer BT474 cells, and mouse embryonic fibroblasts (MEF) were cultured in DMEM medium (10% CO_2_). Media were supplemented with 10% fetal bovine serum (Life Technologies, Grand Island, NY, USA), 1% glutamine, 2% pyruvate, and an antibiotics mixture (Bio-Lab, Jerusalem, Israel). JNK1 KO and JNK2 KO MEFs were kindly provided by Dr. Michael Karin (University of California, San Diego, CA, USA), RIPK3 KO MEF immortalized with SV-40 large T-antigen by Drs. Seongmin Yoon and David Wallach (Weizmann Institute of Science, Rehovot, Israel), Bid KO MEFs by Dr. Stanley J. Korsmeyer (Harvard Medical School, Boston, MA, USA), and MLKL KO MEF by Dr. Zheng-Gang Liu (National Cancer Institute, NIH, Bethesda, MD, USA).

### Reagents

Normal human serum (NHS) prepared from the peripheral blood of healthy individuals (3H Biomedical AB, Uppsala, Sweden) served as a source for complement. Its use was approved by the Ethics Committee of Tel Aviv University. Heat-inactivated NHS (HIS) was prepared by incubation of NHS for 45 min at 56°C and served as a negative control to complement activation. NHS and HIS were kept at −70°C until used. Purified human C9 and C9-depleted human serum (C9D-NHS) were purchased from Complement Technology, Inc. (Tyler, TX, USA). The Alexa Fluor 546 protein labeling kit was from Molecular Probes, Inc. (Eugene, OR, USA). C9 was labeled with Alexa Fluor 546 according to the manufacturer’s instructions (38). Mouse anti-RIPK3 and goat anti-RIPK1 were purchased from Santa Cruz Biotechnology (CA, USA). Rat anti-MLKL antibody was purchased from Milipore (Bedford, MA, USA). Mouse anti-actin antibody was purchased from Chemicon (Temecula, CA, USA). Peroxidase-conjugated rabbit anti-goat IgG and goat anti-mouse IgG, FITC-conjugated donkey anti-goat IgG and goat anti-mouse IgG were from Jackson Immunoresearch Laboratories (West Grove, PA, USA). Secondary antibodies for immunofluorescence analysis: Donkey anti-mouse Alexa Fluor 488 and Donkey anti-goat Alexa Fluor 546 were purchased from Thermo Fisher Scientific (Rochester, NY, USA). Monoclonal mouse anti-FLAG antibody and the reagents Nec-1 and propidium iodide (PI) were from Sigma-Aldrich (St. Louis, MO, USA). GSK’872 and Nec-1s (7-Cl-O-Nec-1) were from Merck. NSA was purchased from Abcam (Cambridge, MA, USA) and GW806742X was from Adipogene (San Diego, CA, USA).

### Complement-Dependent Cytotoxicity (CDC) Assay

Human cancer cells were treated for 30 min on ice with rabbit anti-K562 antibodies (prepared in the animal facilities of Tel Aviv University after receiving the approval of the Animal Ethics Committee) diluted in HBSS buffer (Sigma). The rabbit antiserum prepared after immunization with K562 cells reacted well with common human epitopes on diverse human carcinoma cells and efficiently activated their CDC. NHS was added (as a source of complement) to a final concentration of 50% and incubation was continued for 60 min at 37°C. The sublytic and lytic doses of the antibodies were determined in a titration experiment. Cytotoxicity assays with mouse fibroblasts were performed with diluted NHS only (at lytic or sublytic serum dilutions), since NHS contains natural complement-fixing and anti-mouse cell antibodies. Adherent cells were collected by trypsinization (Biological Industries, Israel) prior to CDC testing. CD was detected by PI (1 µg/ml) inclusion and trypan blue (0.02%) exclusion. Cells were analyzed by a FACScan (Becton Dickinson, San Jose, CA, USA) or microscopically, respectively. Flow cytometry data were analyzed by WinMDI 2.8 software.

To improve the quantification of differences between cytotoxicity values and to calculate the inhibition percentage of CDs, the cytotoxicity percentage was converted into *y*/1 − *y* in which 100y = the percentage of CDs (39). Thus, at a percentage cytotoxicity of 50%, *y* = 0.5 and *y*/1 − *y* = 0.5/(1 − 0.5) = 1.

### Transfection of Plasmids and siRNA

Receptor-interacting protein kinase 3-shRNA in pSuperior. puro and Flag-RIPK3 and Flag-RIPK3-K50A (a kinase-dead form of RIPK3) in pcDNA3.1 were kindly provided by Liming Sun and Xiaodong Wang (National Institute of Biological Sciences, Beijing, China). WT and kinase-dead mutant RIPK1 (RHIM domain-ALAA) in pEGFP-N1 were kindly provided by Francis Chan (University of Massachusetts, Worcester, MA, USA).

To prepare RIPK1 shRNA, sense and antisense hairpin oligonucleotides of RIPK1, selected from the RNAi consortium (TRC) library (ID: TRCN0000197069 and TRCN0000199741), were ordered from Sigma-Aldrich (St. Louis, MO, USA). The DNA was ligated into AgeI/EcoRI-digested pLKO.1 plasmid. Following cloning, the RIPK1 shRNA sequence was confirmed by sequencing. pLKO.1 empty vector and a vector containing a scrambled shRNA sequence were obtained from Chen Luxenburg, Tel Aviv University. MLKL siRNA pool was purchased from Dharmacon (Lafayette, LA, USA): sense:5′-GGAAGGAGCUCUCGCUGUU-3′, 5′-GAGCAACGCAUGCCUGUUU-3′,5′-AAUAGAAGCUUCACUGAGA-3′,5′-GAGAUGAAAUACUGCAAGA-3′ and scrambled control: sense:5′-UAAGGCUAUGAAGAGAUAC-3′, 5′-AUGUAUUGGCCUGUAUUAG-3′,5′-AUGAACGUGAAUUGCUCAA-3′,5′-UGGUUUACAUGUCGACUAA-3′.

K562 cells were transfected with plasmid DNA using electroporation (250 V, 14 ms) in a sterile 0.4 cm electroporation cuvette (Cell Projects, Kent, UK). HEK-293T cells were transfected by using Lipofectamine 2000 (Invitrogen-Life Technologies, Grand Island, NY, USA) according to the manufacturer’s instructions.

### Co-Immunoprecipitation (co-IP)

#### RIPK1–RIPK3 co-IP

Cell lysates were prepared by treating cells with 0.2% NP-40 in 20 mM Tris–HCl pH 7.5, 150 mM NaCl, 1 mM EDTA, 3 mM sodium fluoride, 1 mM sodium orthovanadate, 1 mM β-gylcerophosphate, and 10% glycerol. Protein-A/G Agarose beads (Santa Cruz) were precoated with anti-RIPK1 antibodies in PBS and added to cell lysates for 1 h at 4°C (on a rotating platform). After having been washed with lysis buffer, the beads were mixed in reducing SDS-PAGE sample buffer, warmed to 95°C for 5 min, and the bound proteins were analyzed by SDS-PAGE and Western blotting with anti-RIPK1 and anti-RIPK3 antibodies (9).

#### C9-MLKL co-IP

Treated cells were lysed in 100 mM Tris, pH 7.5, 0.77% Triton X-100, and 10 mM EDTA buffer. Cell lysates were precipitated with anti-C9 antibody-coated Protein-A/G Agarose beads as described above. Bound C9 and MLKL were detected as described above.

#### MLKL-C5b-9 co-IP

Treated cells were lysed in 100 mM Tris, pH 7.5, 0.77% Triton X-100, and 10 mM EDTA buffer. Cell lysates were precipitated with anti-MLKL antibody-coated Protein-A/G Agarose beads as described above. Bound proteins were eluted with 0.2 M glycine, pH 2.3 for 10 min, followed by neutralization with bicarbonate buffer pH 8.9. To detect MLKL, samples were analyzed by SDS-PAGE and Western blotting by using anti-MLKL antibodies. To detect bound C5b-9, the samples were analyzed by ELISA. Samples were attached overnight at 4°C to wells of a 96-well plate (Nunc, Rochester, NY, USA). After having been blocked with 1% BSA in TBS, the wells were treated with anti-C5b-9 aE-11 antibody for 2 h at room temperature and developed with peroxidase-labeled goat anti-mouse IgG for 1 h at room temperature, and with TMB Substrate (SouthernBiotech) that was added to each well. The reactions were stopped by adding TMB Stop Solution. The absorbance was measured with an ELISA Plate Reader at 450 nm.

### Confocal Immunofluorescence Microscopy

Cells were treated with a sublytic dose of antibody and NHS, HIS, or C9D-NHS supplemented with C9-AF546 at 37°C. Next, the cells were fixed with 2% paraformaldehyde (10 min at room temperature), washed with buffer containing 0.5% bovine serum albumin and 0.1% saponin, and then blocked with blocking buffer containing 0.5% BSA, 0.1% saponin, and 10% fetal calf serum. Next, the cells were labeled with the primary antibody diluted in blocking buffer for 1 h at room temperature or overnight at 4°C, washed, and then labeled with a secondary fluorescently tagged antibody diluted in blocking buffer for 1 h at room temperature. The cells were imaged directly on a cover slip under a Zeiss Laser Scanning Confocal TCS II Microscope (Oberkochen, Germany). Images and merged images were obtained with LSM software (Carl Zeiss, Germany).

### Fluorescence Resonance Energy Transfer (FRET) Analysis

Cells transfected with Flag-RIPK3 plasmid were treated with a sublytic dose of anti-K562 antibody and NHS (5 min at 37°C). Then, they were fixed with 2% formaldehyde for 5 min and permeabilized with 0.5% saponin. Next, the cells were labeled with primary goat anti-RIPK1 or mouse anti-FLAG antibodies, followed by AF546-labeled donkey anti-goat IgG or AF488-labeled donkey anti-mouse IgG secondary antibodies, respectively. The cells then were imaged directly on a cover slip under a Zeiss LSM 510, Meta Microscope. Square ROI over the area of the cell was photobleached by 200 pulses to eliminate the acceptor (AF 546) while monitoring the emission channel of the donor (AF488) using a high-intensity Ar 561 nm laser. Images were taken before and after bleaching. Image processing and analysis were performed with ImageJ software.

FRET efficiency (E) was calculated as follows:

$$\begin{array}{c} \text{E}=\text{(FI of donor after bleaching}-\text{FI of donor before bleaching)}\\ \;/\text{FI of donor after bleaching }(\text{FI, fluorescence intensity}). \end{array}$$

### Statistical Analysis

Student’s *t*-test was performed to determine the statistical significance of differences between two data sets. Multiple group comparison was calculated by one-way ANOVA. Two-way ANOVA was performed to compare two groups, a tested reagent, and the interaction between them. SPSS 24.0 software was used. Statistical significance was proposed when the *P*-value was smaller than 0.05.

## Results

### A Role for RIPK1 in CDC

**Figure 1:**
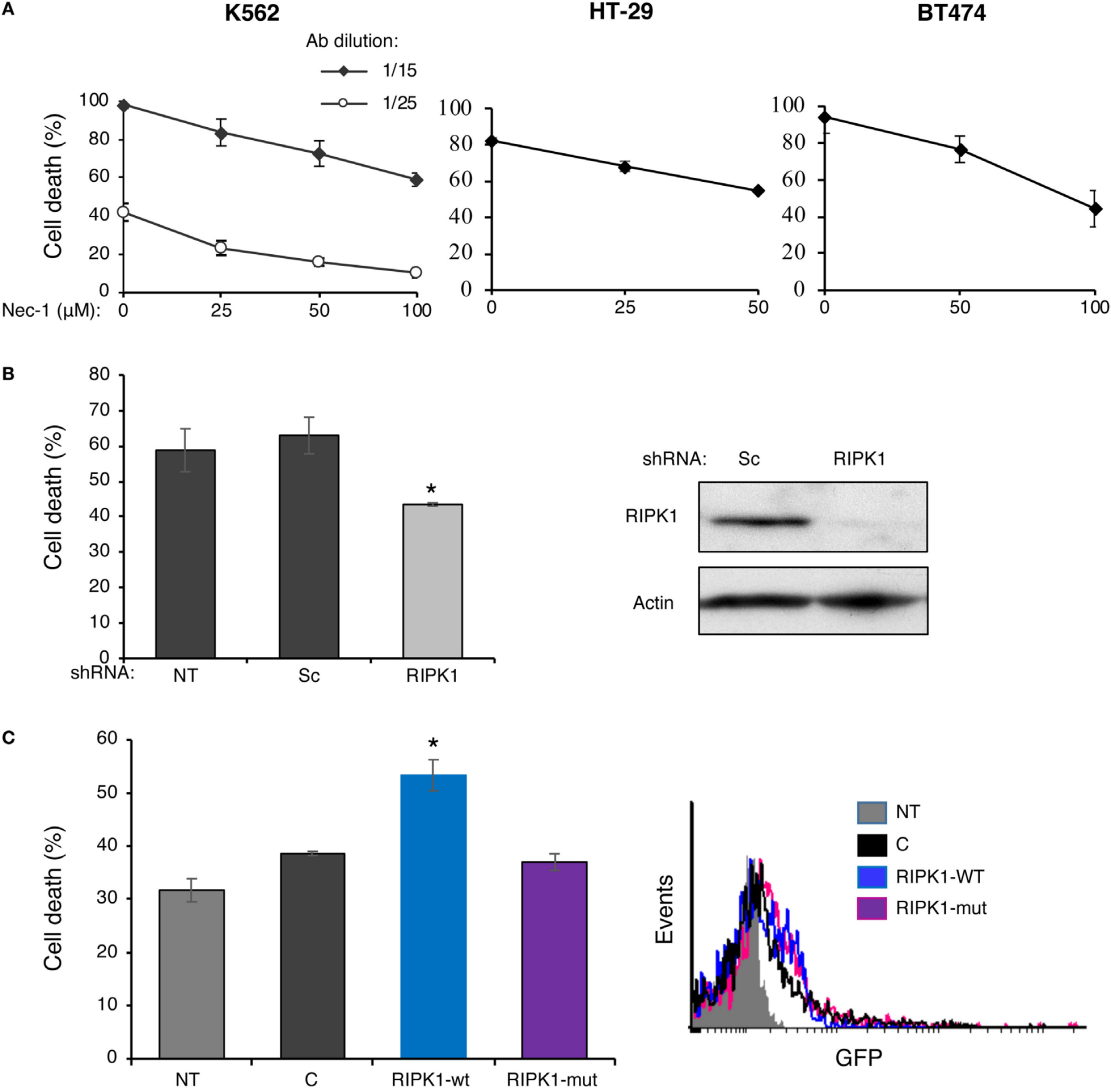
Complement C5b-9 induces receptor-interacting protein kinase 1 (RIPK1)-dependent necrosis. **(A)** K562, HT-29, or BT474 cells were treated with necrostatin-1 (Nec-1) or with DMSO (0) as control for 1 h at 37°C. Cell death (CD) by antibody (30 min at 4°C) and complement (1 h at 37°C) was performed as described under Section “Materials and Methods.” The experiment with K562 cells was performed with two antibody (Ab) dilutions. The percentage of CD was analyzed by propidium iodide inclusion. Results of three independent experiments are expressed as the mean percentage of CD ± SD. The percentage of CD by Nec-1, antibody, and HIS was 3–7% (negative controls). Statistical analysis showed that Nec-1 significantly inhibited CD (one-way-ANOVA, *P* < 0.01). **(B)** K562 cells transfected for 48 h with RIPK1-shRNA, a scrambled (Sc) shRNA, or not transfected (NT) were treated with antibody and complement and the percentage of CD was determined as described in panel **(A)**. Transfection with RIPK1-shRNA significantly inhibited the CD relative to the scrambled shRNA (*t*-test, *P* < 0.01). The RIPK1 expression level (relative to actin) in the transfected cells was assessed by Western blotting analysis. A representative blot is shown. **(C)** K562 cells were transfected with the GFP-tagged plasmids: wild-type RIPK1 (RIPK1-wt), RHIM-ALAA mutant (RIPK1-mut), an empty vector control (C), or were NT. After 52 h, the cells were treated with antibody and complement and the CD was determined as above. Transfection with RIPK1-wt significantly enhanced the CD relative to empty vector and RIPK1-mut (*t*-test, *P* < 0.01). Transfection efficiency was determined by analyzing the GFP expression by flow cytometry. A representative histogram is shown. All results represent three independent experiments.

Receptor-interacting protein kinase 1 kinase activity is required for necroptosis induced *in vitro* by Fas, TNFα, and TRAIL death receptors as well as other inducers. In order to determine whether RIPK1 plays a role in CDC, we first determined how Nec-1 affects the sensitivity of K562, HT-29, and BT474 cells to treatment with antibody and complement. Inhibition of the kinase activity of RIPK1 by Nec-1 was shown to block death receptor-induced necroptosis in different cellular models (12, 40). Cells were pretreated with Nec-1 and then subjected to a CDC assay. As shown in Figure 1A, Nec-1 markedly reduced CDC in a concentration-dependent manner in the three cell types, suggesting a role for RIPK1 in the C5b-9-induced signaling that leads to necrotic CD. Transient transfection of K562 cells with a RIPK1 shRNA plasmid markedly lowered the expression of RIPK1 protein and reduced cell sensitivity to CDC (Figure 1B). Similarly, HEK-293T cells transfected with RIPK1 shRNA were partially resistant to CDC (Figure S1 in Supplementary Material). On the other hand, overexpression of RIPK1 in K562 cells by transient plasmid transfection enhanced cell sensitivity to CDC (Figure 1C). During TNFα-induced necroptosis, RIPK1 interacts with RIPK3 through RHIM (RIP homotypic interaction motifs) (29, 31, 32). As shown here, unlike the wild-type (WT) RIPK1, overexpression of the RHIM-ALAA RIPK1 mutant in K562 cells failed to upregulate CDC (Figure 1C).

### A Role for RIPK3 in CDC

**Figure 2:**
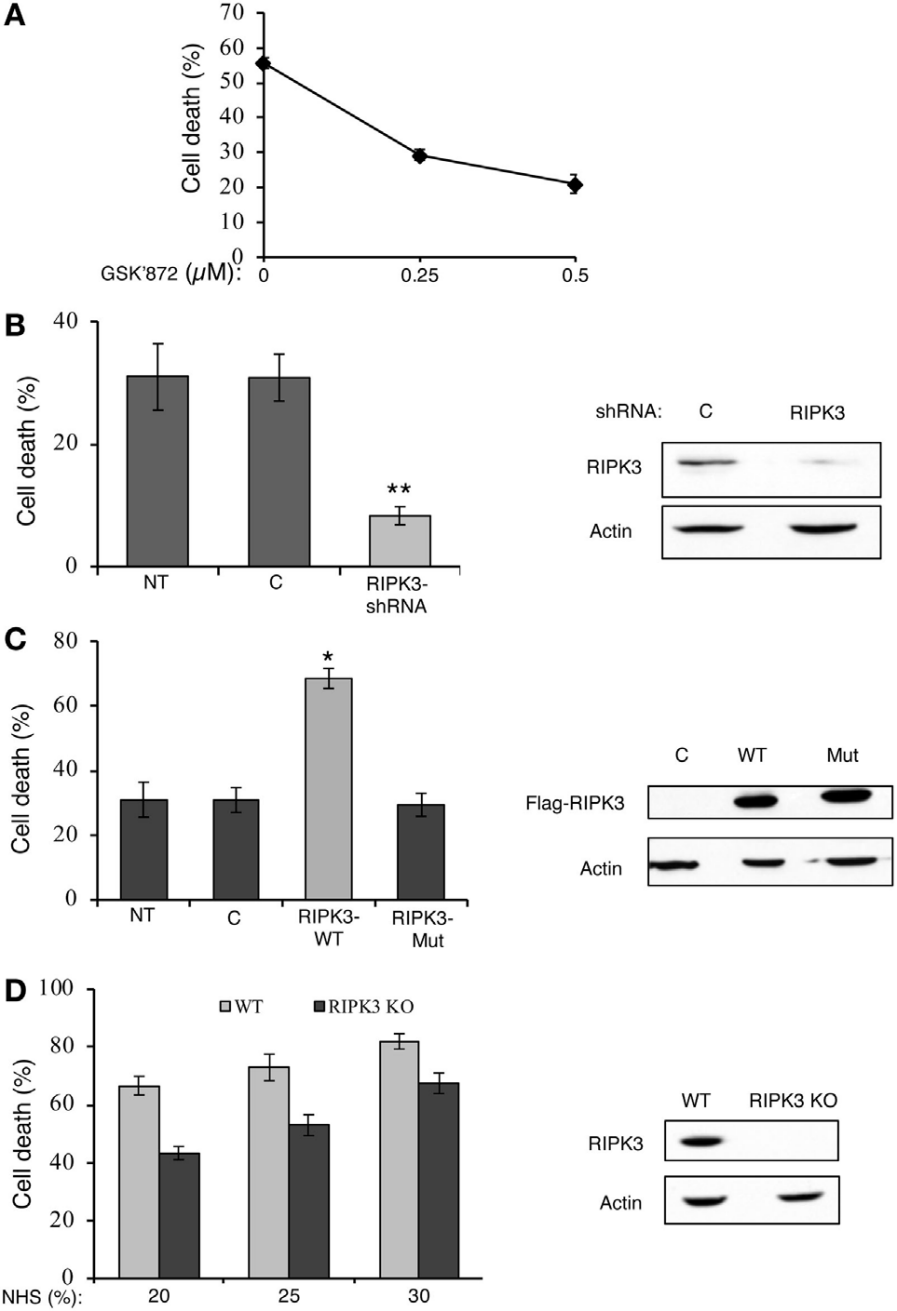
Receptor-interacting protein kinase 3 (RIPK3) contributes to complement-induced cell death (CD). **(A)** K562 cells were pretreated with GSK’872 or with DMSO as control for 1 h at 37°C and then treated with antibody and complement. The percentage of CD was analyzed by propidium iodide inclusion. Statistical analysis showed that GSK’872 significantly inhibited CD (one-way-ANOVA, *P* < 0.01). **(B)** K562 cells were transfected with a RIPK3-shRNA plasmid or an empty vector **(C)** as control. After 48 h, the cells were treated with antibody and complement and CD was quantified. Transfection with RIPK3-shRNA significantly reduced CD relative to empty vector (*t*-test, *P* < 0.01). RIPK3 knockdown was confirmed by Western blotting analysis of cell lysates (shown on the right). **(C)** K562 cells were transfected with plasmid DNA expressing Flag-tagged wild-type (WT) RIPK3 (RIPK3-wt), RIPK3 kinase-dead mutant (RIPK3-mut), or with its empty vector **(C)**. After 30 h, the cells were treated with antibody and complement and CD was determined. Transfection with RIPK3-wt significantly enhanced CD relative to empty vector and RIPK3-mut (*t*-test, *P* < 0.05). To confirm the expression of the flagged proteins, cell lysates were subjected to Western blotting analysis with anti-Flag and anti-actin antibodies (shown on the right). **(D)** WT and RIPK3 KO mouse embryonic fibroblasts (MEFs) were treated with normal human serum (NHS) (20, 25, or 30%) for 1 h at 37°C. The percentage of CD was determined by trypan blue exclusion. Statistical analysis showed that RIPK3 KO MEF is significantly less sensitive to complement-dependent cytotoxicity than is WT MEF (two-way-ANOVA, *P* < 0.001). Yet, the two cell types reacted similarly to increasing concentrations of complement (two-way-ANOVA, *P* = 0.148). Lysates of WT and RIPK3 KO MEFs were analyzed by Western blotting with anti-RIPK3 and anti-actin antibodies (shown on the right). Results are expressed as mean percentage of CD ± SD. Data shown represent at least three independent experiments. NT, not transfected cells.

Receptor-interacting protein kinase 3 is also a key regulator of necroptosis (27–29). GSK’872, a potent and selective RIPK3 kinase inhibitor, is known to prevent necroptosis induced by TNFα, viruses, or through TLR3 (41). The effect of GSK’872 on the death of K562 cells treated with complement was tested. As shown in Figure 2A, pretreatment with GSK’872 protected cells from CDC. Consistently, knockdown of RIPK3 expression in K562 cells by transfection with a RIPK3-shRNA plasmid DNA also diminished CDC (Figure 2B). RIPK3 overexpression potentiates TNFα-induced necroptosis and its expression in different cell lines correlates with their necrotic sensitivity (27). Similarly, overexpression of RIPK3 in K562 cells induced elevated CDC levels (Figure 2C). Unlike the WT RIPK3, overexpression of the RIPK3 kinase-dead mutant, K50A, did not increase CDC. A kinase-defective RIPK3 mutant failed to induce necroptosis in TNFα-treated HT-29 and Jurkat cells (27, 29). Fibroblasts from RIPK3 knockout mice are resistant to necroptosis induced by TNFα (27) or through TLR3 (41). To investigate the relevance of RIPK3 to CDC in mouse cells, the CD of RIPK3 KO and WT mouse fibroblasts by complement was compared. As shown in Figure 2D, RIPK3 KO mouse fibroblasts were found to be significantly less sensitive to CDC than were WT mouse fibroblasts. These results indicated that like in necroptosis, RIPK1 and RIPK3 play a role in CDC.

### Complement Induces RIPK1–RIPK3 Interaction

**Figure 3:**
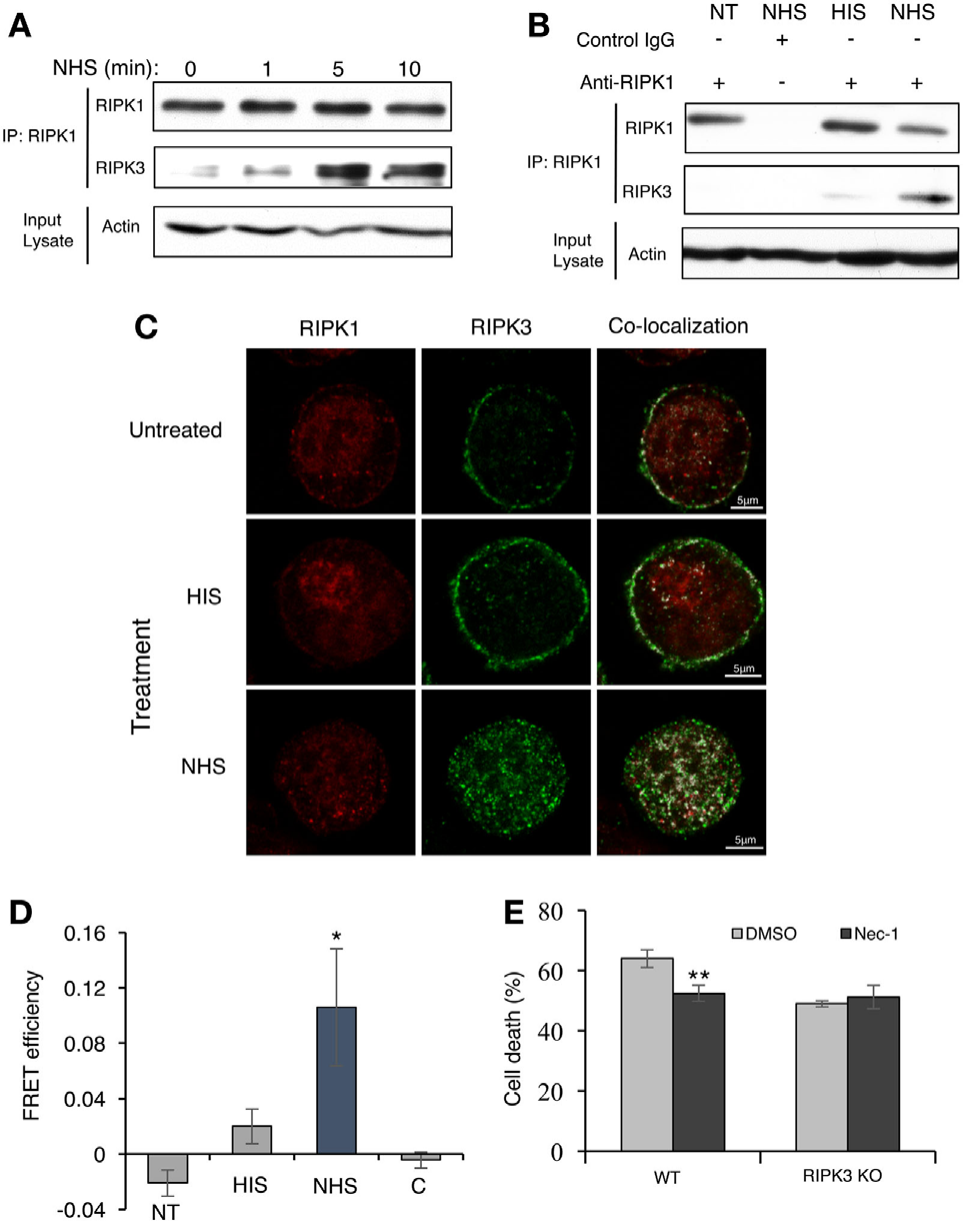
Complement triggers receptor-interacting protein kinase 1 (RIPK1) and receptor-interacting protein kinase 3 (RIPK3) complex formation. **(A)** Flag-RIPK3 transfected K562 cells were treated with a sublytic dose of antibody and complement for the indicated times. Cell lysates were immunoprecipitated with anti-RIPK1 antibody. Immunocomplexes were subjected to Western blotting with anti-RIPK1 or anti-Flag antibodies. Aliquots of cell lysates (Input lysate) were also analyzed for the amount of actin. **(B)** Flag-RIPK3 transfected K562 cells were treated with a sublytic dose of antibody and complement for 10 min at 37°C. Cell lysates were immunoprecipitated with anti-RIPK1 antibody or with goat IgG as control. Controls included cells treated with HIS (heat-inactivated serum) or untreated cells [not transfected (NT)]. RIPK1, RIPK3, and actin were detected as above. **(C)** Flag-RIPK3 K562 cells were treated with a sublytic dose of antibody and then with complement (or HIS) for 10 min at 37°C. Cells were fixed and permeabilized. RIPK3 was detected by using anti-Flag antibody followed by AF488-labeled secondary antibody. Endogenous RIPK1 was labeled with anti-RIPK1 antibody followed by AF546-labeled secondary antibody. After analysis by confocal microscopy, the RIPK1 and RIPK3 locations were merged by the Co-localization plugin in ImageJ software. **(D)** Flag-RIPK3 K562 cells were treated for 5 min with a sublytic dose of antibody and complement. Then, they were fixed and permeabilized. Prior to imaging, cells were treated with goat anti-RIPK1 or mouse anti-FLAG antibodies, followed by AF546-labeled donkey anti-goat IgG or AF488-labeled donkey anti-mouse IgG secondary antibodies, respectively. Square ROI was photobleached and images were taken before and after bleaching. Shown are fluorescence resonance energy transfer (FRET) efficiencies normalized to the bleaching percentage of the acceptor. Control (C) is the FRET efficiency outside the bleached regions [in normal human serum (NHS)-treated cells] *P < 0.05 relative to HIS. **(E)** WT and RIPK3 KO mouse embryonic fibroblasts were pretreated with Nec-1 (50 µM) or with DMSO as control and then treated with NHS for 1 h at 37°C. The percentage of cell death was determined by trypan blue inclusion. Data were represented as mean ± SD of triplicates. **P < 0.01 relative to DMSO. All experiments were repeated at least three times with similar results.

Activators of necroptosis induce interactions between RIPK3 and RIPK1 and the formation of a pro-necrotic protein complex. Activation of RIPK3–RIPK1 binding by complement C5b-9 was tested. Briefly, K562 cells were stably transfected with Flag-tagged RIPK3 and then treated with a sublytic dose of antibody and complement for 1–10 min. Cell lysates were subjected to immunoprecipitation with anti-RIPK1 antibodies. Subsequently, the immunoprecipitated proteins were analyzed by Western blotting with anti-Flag (RIPK3) antibodies. As shown in Figures 3A,B, RIPK3 co-immunoprecipitated with RIPK1 in lysates of cells exposed to sublytic complement. Binding was not observed or was very low in lysates of untreated cells or cells treated with antibody and heat-inactivated complement (HIS) (Figure 3B).

The locations of RIPK1 and RIPK3 in cells treated with sublytic antibody and complement were analyzed by confocal fluorescence microscopy. Next, Flag-RIPK3-transfected K562 cells were treated with antibody and NHS for 10 min, after which the cells were fixed and RIPK1 and RIPK3 were immunofluorescently labeled and imaged. As shown in Figure 3C, in control cells, RIPK1 was finely dispersed throughout the cytoplasm, whereas most of the RIPK3 was located in the plasma membrane region. After 10 min of treatment with sublytic complement (NHS), most of the RIPK3 translocated from the cell periphery into the cytoplasm, where it formed dotted structures. Interestingly, intense co-localization of RIPK1 and RIPK3 throughout the cells was observed in complement-treated cells but not in control cells. Attempts to identify the localization of RIPK3 clusters to the nucleus, lysosomes, mitochondria, or ER were not successful (Figure S2 in Supplementary Material).

The postulated contact between RIPK1 and RIPK3 was verified by FRET analysis (Figure 3D). K562 cells stably expressing Flag-tagged RIPK3 were treated with a sublytic dose of antibody and complement for 5 min. Then, they were fixed and RIPK1 (AF546) or Flag-RIPK3 (AF488) was labeled with fluorescent antibodies. An area of a cell was photobleached to eliminate the acceptor (AF546) while monitoring the fluorescence intensity of the donor emission (AF488). Depletion of AF546 fluorescence in complement-treated cells resulted in about a 10% increase in the AF488 fluorescence (Figure 3D). FRET was not detected in cells treated with HIS or in untreated control cells.

To confirm that RIPK3 and RIPK1 cooperate in the same activation pathway leading to CDC, the effect of Nec-1 on CDC was examined in cells lacking RIPK3. As shown in Figure 3E, Nec-1 reduced the CD induced by complement in WT mouse fibroblasts. In contrast, Nec-1 did not affect the death of mouse fibroblasts that lack RIPK3. This demonstrates that functional cooperation exists between RIPK1 and RIPK3 in signaling CDC.

### A Role for MLKL in CDC

**Figure 4:**
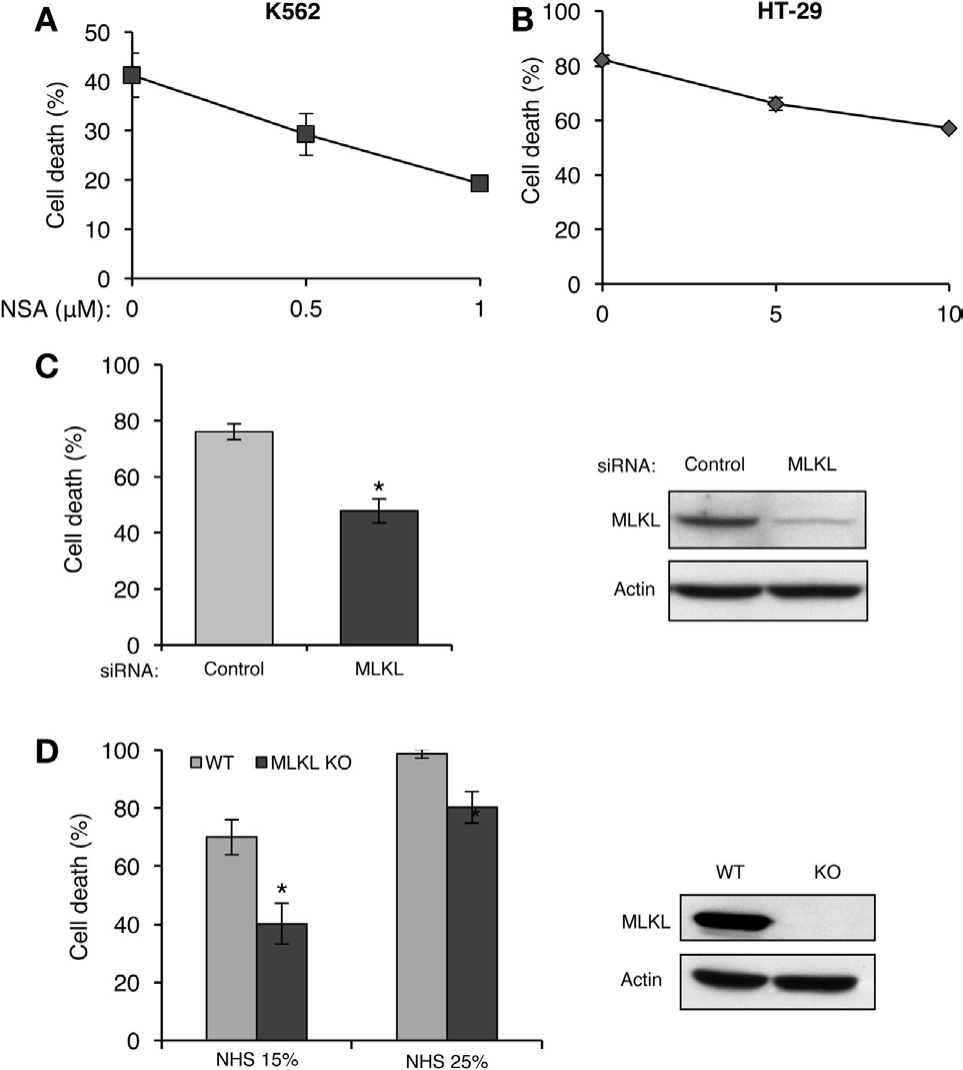
Mixed lineage kinase domain-like protein (MLKL) contributes to complement-induced cell death (CD). **(A,B)** K562 **(A)** and HT-29 cells **(B)** were incubated with necrosulfonamide (NSA) or with DMSO as control for 1 h at 37°C and subsequently with antibody and normal human serum (NHS). Necrotic CD was determined by propidium iodide (PI) inclusion. Results, representing three independent experiments, are expressed as the mean percentage of CD ± SD. Differences between GSK’872 and DMSO (0) treatments were all significant (*P* < 0.05). **(C)** K562 cells were transfected with Smart Pool siRNA targeting MLKL or scrambled siRNA as a control, by electroporation. After 48 h, the cells were treated with antibody and complement for 1 h at 37°C. CD was measured by PI staining and expressed as the mean percentage of CD ± SD and the results represent three independent experiments. *P < 0.001 relative to control. Cell lysates, collected 48 h posttransfection, were subjected to Western blotting with anti-MLKL or anti-actin antibodies (shown on the right). **(D)** WT and MLKL KO mouse embryonic fibroblasts were treated with NHS (15 or 25%) for 1 h at 37°C. The CD percentage was measured by trypan blue inclusion. Results are the mean percentage of death ± SD. *P < 0.001 relative to WT control. Cytoplasmic aliquots were subjected to Western blot analysis of MLKL and actin levels (shown on the right).

Receptor-interacting protein kinase 3 activates necroptosis through MLKL (33–35). The finding that complement triggers a RIPK3-dependent necrotic CD suggests that MLKL is also involved in CDC. K562 and HT-29 cells were pretreated with NSA and then subjected to CDC by using lytic doses of antibody and complement. As shown in Figures 4A,B, pretreatment with NSA reduced CDC in a concentration-dependent manner in the two cell types. Similarly, knocking down of MLKL in K562 cells with siRNA reduced CD (Figure 4C). Mouse fibroblasts that lack MLKL were also found to be more resistant to CD compared with WT fibroblasts (Figure 4D).

**Figure 5:**
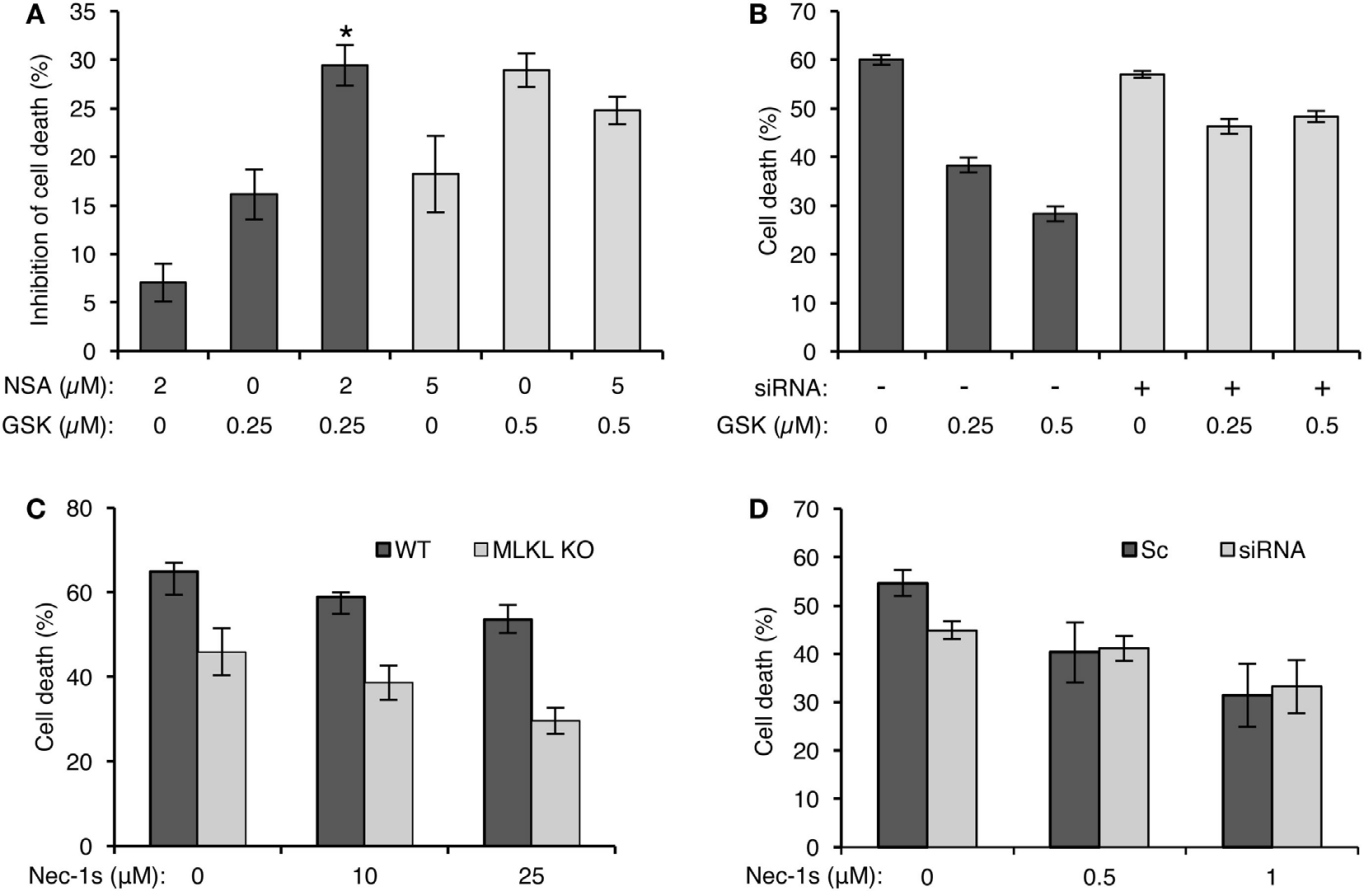
Mixed lineage kinase domain-like protein (MLKL) cooperates with RIPK3 during complement-dependent cytotoxicity (CDC) but not with receptor-interacting protein kinase 1 (RIPK1). **(A)** K562 cells were pretreated with GSK’872 and/or necrosulfonamide (NSA) in the indicated concentrations or with DMSO as control (0), for 1 h at 37°C, and then treated with antibody and complement for 1 h min at 37°C. The percentage of cell death (CD) was measured by propidium iodide (PI) inclusion. Results are expressed as the mean percentage of the inhibition of CD ± SD and represent three independent experiments. *P < 0.01 relative to pretreatment with 0.25 µM GSK’872 without NSA or to 2 µM NSA without GSK’872. [% Inhibition = (% Death of Control − % Death of Treatment)/%Death of Control × 100]. **(B)** K562 cells were transfected with Smart Pool siRNA targeting MLKL (siRNA) or scrambled siRNA (Sc) as a control by electroporation. After 24 h, the cells were treated with GSK’872 at the indicated concentrations (or DMSO as control, 0) and then with antibody and complement for 1 h at 37°C. CD was measured as above. Statistical analysis showed that CDC of MLKL knocked down cells is significantly less sensitive to GSK’872 than is CDC of cells treated with a scrambled siRNA (two-way-ANOVA, *P* < 0.001). **(C)** WT and MLKL KO mouse embryonic fibroblasts (MEFs) were treated with necrostatin-1 (Nec-1s), a selective RIPK1 inhibitor, in increasing concentrations, or with DMSO as control, for 1 h followed by incubation with 10% normal human serum (NHS). The lysis percentage was determined by PI inclusion. Results are presented as the mean percentage of lysis ± SD. Statistical analysis showed that MLKL KO MEF is as sensitive to CDC as WT MEF (two-way-ANOVA, *P* = 0.538). **(D)** MLKL-silenced K562 cells were treated with Nec-1s at the indicated concentrations and then with antibody diluted 1:18 or 1:15 for scrambled or siRNA-treated cells, respectively, followed by complement (NHS, 50%). CD was measured by PI staining, expressed as a calculated mean percentage ± SD, representing three independent experiments. Statistical analysis showed that CDC of MLKL knocked down cells is as sensitive to Nec-1 as CDC of cells treated with a scrambled siRNA (two-way-ANOVA, *P* = 0.104).

To determine whether MLKL cooperates with RIPK3 in activating complement-mediated necrotic CD, we investigated the effect of NSA on the CDC of cells with inactive RIPK3 (GSK’872-treated cells). At lower inhibitor concentrations (2 µM NSA and 0.25 µM GSK’872), their combined effect on the inhibition of CD was additive (Figure 5A). However, combining these two inhibitors at higher concentrations (5 µM NSA and 0.5 µM GSK’872) did not result in an additional effect on the inhibition of CD than each inhibitor did alone. This indicates that RIPK3 and MLKL act during CDC in concert, along the same pathway. Further support to this claim was gained by results (Figure 5B) showing that GSK’872 failed to significantly reduce CD in MLKL knocked-down cells. Next, the involvement of RIPK1 in the RIPK3/MLKL-dependent pathway of CDC was examined by testing the effect of Nec-1s on the CDC of MLKL KO mouse fibroblasts. Cells were pretreated with Nec-1s for 1 h and then treated with NHS. Surprisingly, Nec-1s inhibited the CDC of MLKL KO and WT mouse fibroblasts to a similar degree (Figure 5C). Similar results were observed when Nec-1s’ inhibitory effect was tested on the CDC of MLKL-silenced and control K562 cells (Figure 5D), indicating that RIPK1’s pro-necrotic effect during CDC is MLKL independent and that MLKL cooperates only with RIPK3.

### MLKL, RIPK1, and RIPK3 Interact with Complement C5b-9

**Figure 6:**
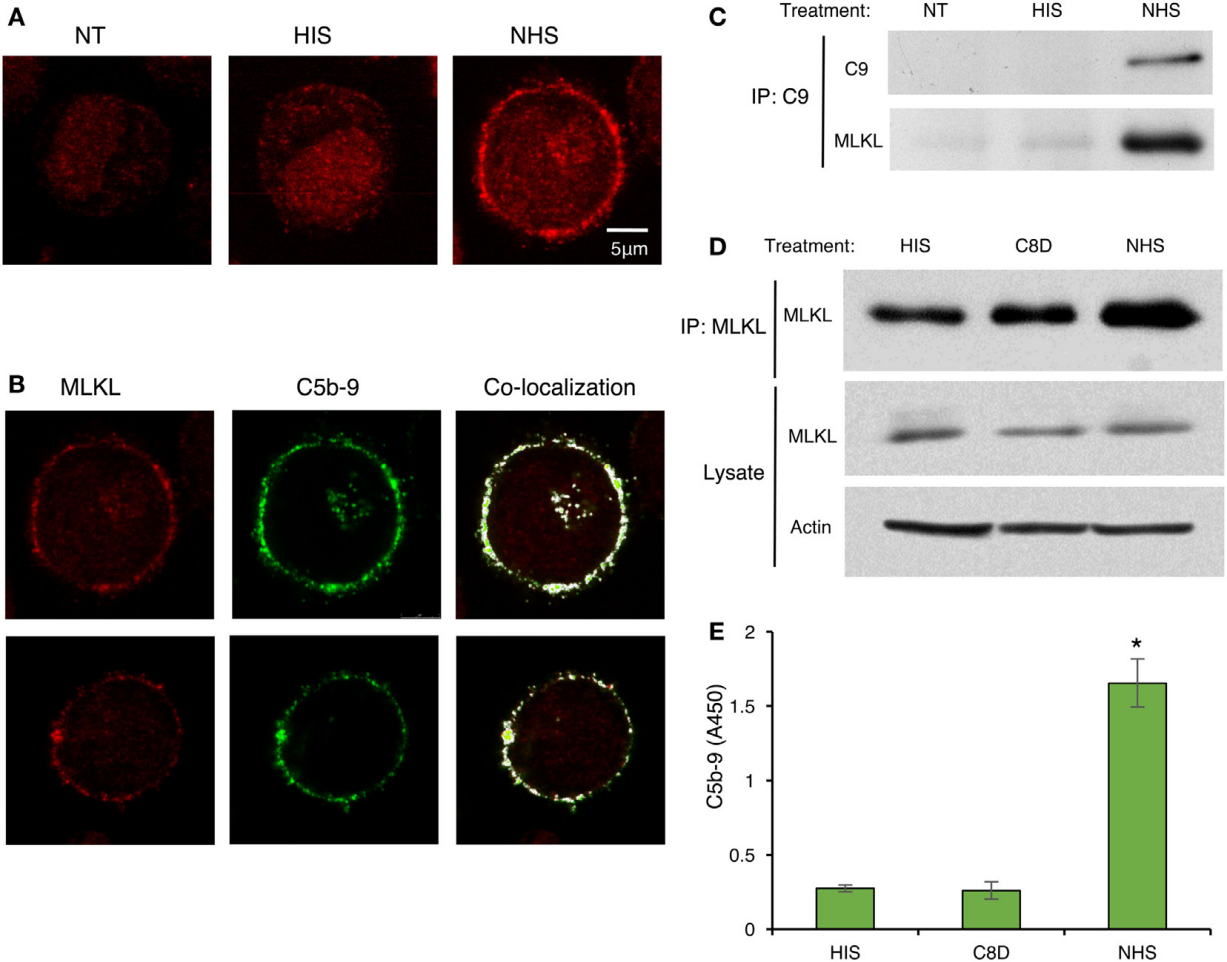
C5b-9 deposition triggers mixed lineage kinase domain-like protein (MLKL) translocation to the plasma membrane and C5b-9-MLKL interaction. **(A)** K562 cells were treated with a sublytic dose of antibody and with complement [normal human serum (NHS)] or HIS for 10 min at 37°C. After fixation and permeabilization, MLKL was stained with anti-MLKL antibody and AF546-labeled secondary antibody. Representative confocal microscope images are presented. NT, non-treated cells. **(B)** K562 cells were treated with antibody and complement as above. In addition to MLKL labeling, C5b-9 was detected with an anti-C5b-9 antibody (AE-11) and with AF488-labeled secondary antibody. MLKL and C5b-9 locations were merged by the Co-localization plugin in ImageJ software. Representative cells (from three independent experiments) show the co-localization of MLKL with C5b-9 in the plasma membrane region. **(C–E)** K562 cells were treated with complement (NHS) as above and cell lysates were prepared. Controls included HIS and C8-depleted human serum (C8D). **(C)** Cell lysates were subjected to immunoprecipitation with anti-C9 antibody followed by Western blot analysis with anti-C9 or anti-MLKL antibodies. **(D)** MLKL was immunoprecipitated from cell lysates with an anti-MLKL antibody. MLKL was detected by Western blotting. MLKL and actin were also quantified in whole-cell lysates (Lysates). **(E)** Proteins immunoprecipitated from cell lysates with anti-MLKL antibodies, as in **(D)**, were eluted from the beads and the amount of C5b-9 was quantified by ELISA. Results are expressed as adsorption at 450 nm (A450). *P < 0.05 relative to C8D or HIS treated cells (*t*-test).

Recent studies have suggested that activated MLKL can interact with the cell membrane to form membrane pores or channels and, thus, MLKL disrupts the membrane (36, 42–45). We hypothesized that during CDC, MLKL interacts with C5b-9, the membranolytic complex of complement. To investigate the plausible binding of MLKL to C5b-9 in the plasma membrane, K562 cells were subjected to sublytic complement and then fixed and immunostained for detection of MLKL and C5b-9 by confocal fluorescence microscopy. In control cells, MLKL was found to be located throughout the cytoplasm and in the nucleus. However, upon exposure to sublytic complement, a large fraction of the MLKL molecules rapidly translocated to the plasma membrane region (Figure 6A). Merging analysis revealed the co-localization of MLKL with C5b-9 at the plasma membrane region. In a few cells, they also appeared in an intracellular compartment previously identified in our studies (46) as the endocytic recycling compartment (ERC) (Figure 6B). We further analyzed by co-IP the interaction of MLKL with C9 and C5b-9 following complement activation on K562 cells. Anti-C9 antibody pulled down MLKL in lysates of cells treated for 10 min with sublytic complement (Figure 6C). MLKL-C5b-9 binding was tested by MLKL pulldown with anti-MLKL antibody. MLKL precipitated to the same extent from lysates of cells treated with antibody and HIS, C8-depleted HS, or NHS (Figure 6D). Next, C5b-9 was quantified in the pulled down material by using an ELISA assay. As shown in Figure 6E, C5b-9 was pulled down only from lysates of cells treated with antibody and NHS.

**Figure 7:**
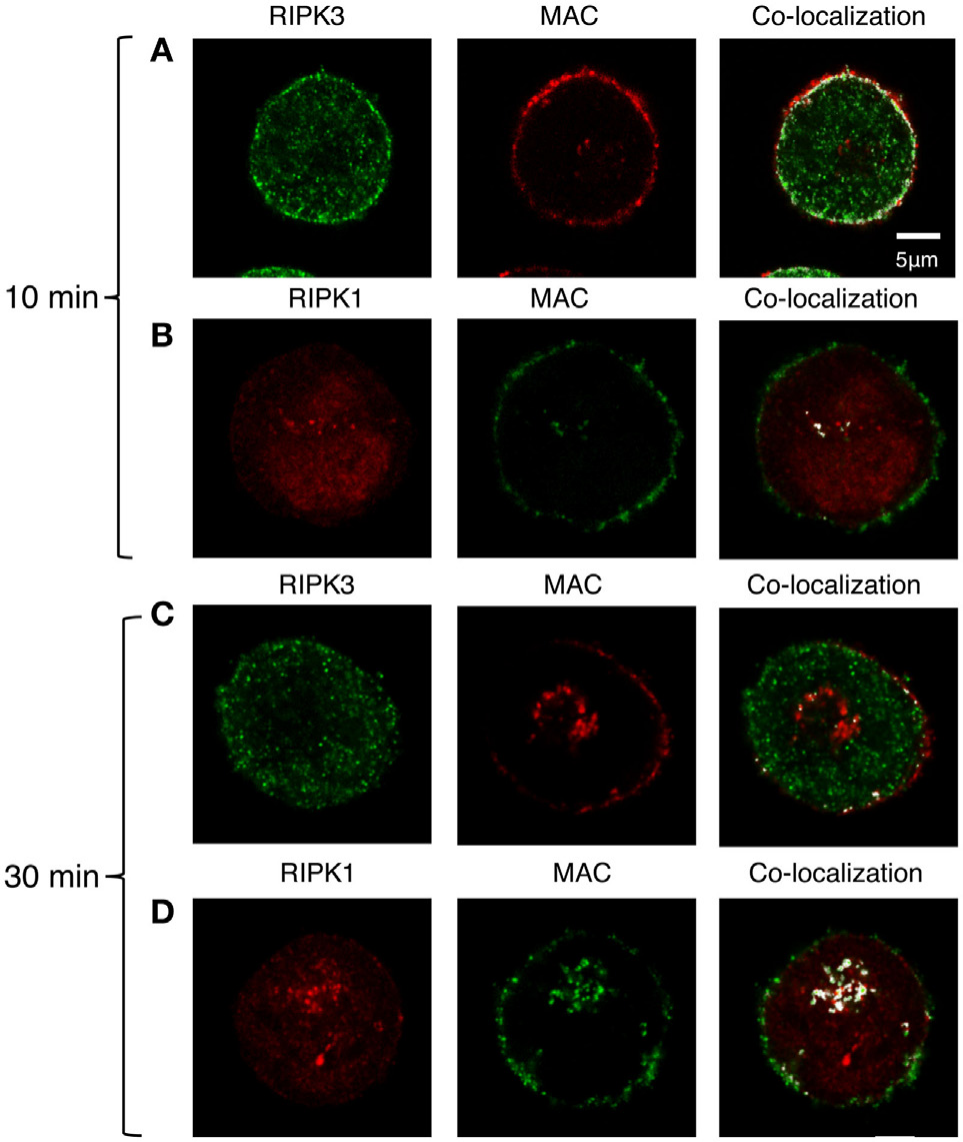
C5b-9 complex co-localizes with receptor-interacting protein kinase 1 (RIPK1) and receptor-interacting protein kinase 3 (RIPK3). **(A,B)** K562 cells expressing Flag-RIPK3 were treated with a sublytic dose of antibody and with C9D-normal human serum (NHS) supplemented with AF546-labeled human C9 **(A)** or with NHS **(B)** for 10 min at 37°C. **(C,D)** Same cells as in panel **(A,B)**, respectively, were then washed and incubated for an additional 20 min in HBSS at 37°C. RIPK3 was detected with anti-Flag antibody and secondary AF488-labeled antibody. C5b-9 was detected with anti-C5b-9 antibody (AE-11) and with secondary AF488-labeled antibody. RIPK1 was labeled with anti-RIPK1 antibody and with AF546-labeled secondary antibody. Representative cells from four independent experiments are shown. Merging of RIPK1 and RIPK3 with C5b-9 locations was performed by the co-localization plugin in ImageJ software.

We next examined whether the activation of RIPK1 and RIPK3 also involves their interaction with the C5b-9 complex. To this end, K562 cells were exposed to a sublytic dose of antibody and either NHS or C9-deficient NHS (C9D-NHS) supplemented with C9-AF546 (46). After 10 min of incubation at 37°C, we observed an extensive co-localization of RIPK3 with C5b-9 in the plasma membrane region (Figure 7A). Almost no interaction between RIPK1 and C5b-9 was observed by fluorescence microscopy (Figure 7B). After 30 min of incubation at 37°C, many C5b-9 complexes were internalized in the cells (Figures 7C,D) as reported earlier (46). At that time, the internalized C5b-9 was co-localized with RIPK1 in an intracellular compartment, probably the ERC (Figure 7D). Low levels of co-localization of C5b-9 with RIPK3 were also observed in the sub-plasma membrane region and in the ERC (Figure 7C).

### Bid and JNK Are Involved in RIPK1/RIPK3/MLKL Signaling during CDC

**Figure 8:**
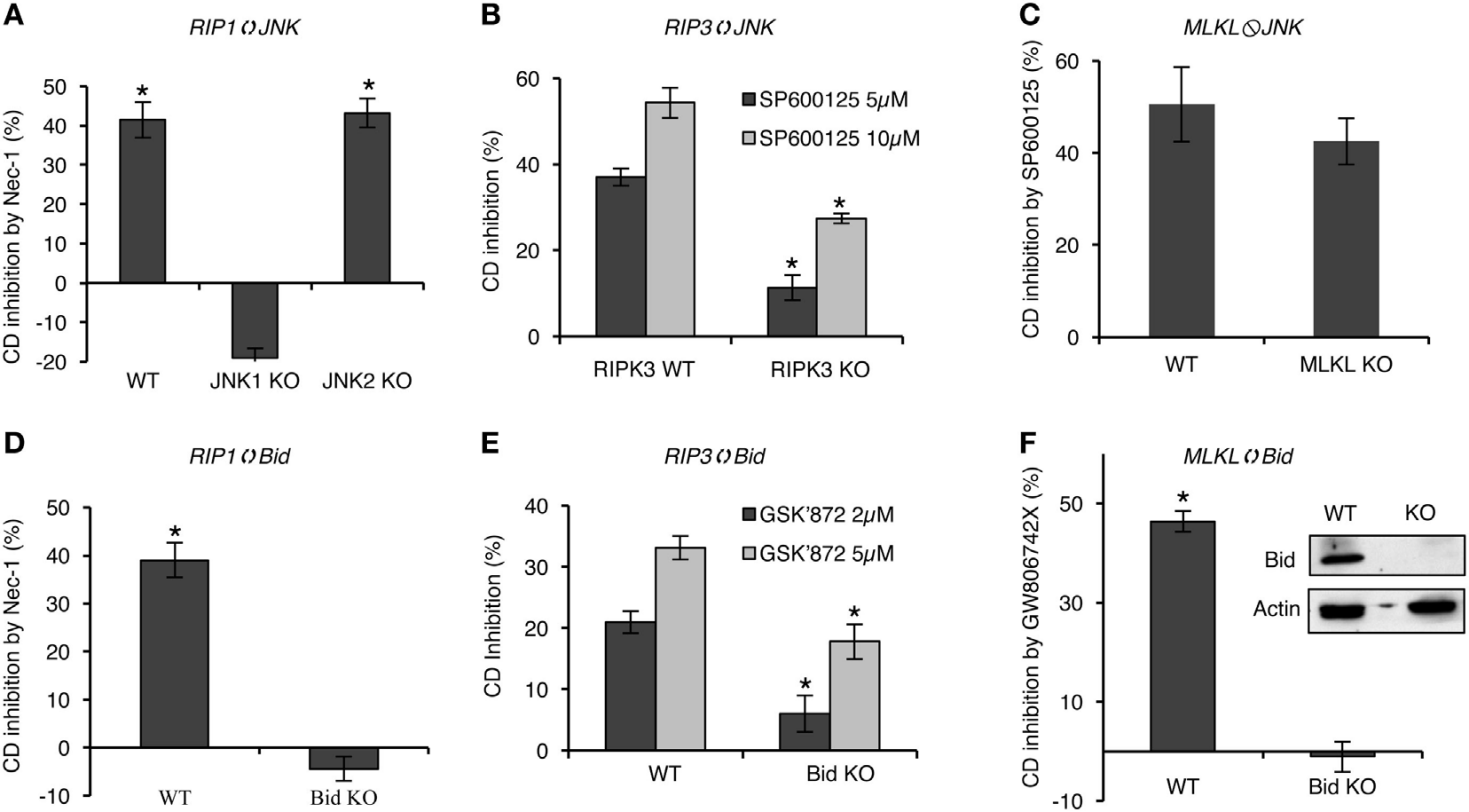
Bid and JNK are involved in receptor-interacting protein kinase 1/receptor-interacting protein kinase 3 (RIPK3)/mixed lineage kinase domain-like protein (MLKL) signaling, leading to complement-induced necrosis. General protocol: cells were pretreated with the indicated inhibitor for 1 h at 37°C and then treated with NHS for 1 h, at 37°C. The percentage of necrotic cell death (CD) and the percentage of CD inhibition by the inhibitor, relative to DMSO, were calculated (after data conversion to *y*/1 − y) as explained in Section “Materials and Methods.” *P < 0.01 relative to DMSO **(A,C,D,F)** or between WT and KO cells **(B,D)** (*t*-test). **(A)** WT, JNK1, or JNK2 mouse embryonic fibroblasts (MEFs) were pretreated with necrostatin-1 (Nec-1) (50 µM) or DMSO as control. **(B)** WT and RIPK3 KO MEFs were pretreated with SP600125 or DMSO as control. **(C)** WT and MLKL KO MEFs were treated with 10 µM SP600125 or DMSO. **(D)** WT or Bid KO MEFs were treated with Nec-1 or DMSO. **(E)** WT or Bid KO MEFs were pretreated with GSK’872 at 2 or 5 µM or with DMSO as control. **(F)** WT or Bid KO MEFs were treated with 1 µM GW806742X or DMSO. All results represent at least three independent experiments.

Bid and JNK were found to promote C5b-9-induced necrotic CD (10, 11). We examined the involvement of Bid and JNK in the complement-induced RIPK1/RIPK3/MLKL-mediated necrotic signaling pathway. The effect of Nec-1 on CDC was studied in Bid KO, JNK1 KO, or JNK2 KO mouse fibroblasts. Nec-1 inhibited the CD of WT and JNK2 KO cells but not of JNK1 KO and Bid KO cells (Figures 8A,D), indicating the involvement of JNK1 and Bid in RIPK1-dependent CDC. The effect of the JNK inhibitor SP600125 on CDC was examined on WT and RIPK3 KO mouse fibroblasts (Figure 8B). SP600125 inhibited the CDC of both RIPK3 KO and WT cells; however, its effect on RIPK3 KO cells was less pronounced, suggesting that the RIPK3-mediated CDC was partially dependent on JNK. Inhibition of CDC by GSK’872 was also lower in Bid KO MEF relative to WT MEF (Figure 8E), suggesting that Bid is involved in RIPK3-dependent CDC. Finally, SP600125 similarly inhibited CDC in wild-type and MLKL KO MEFs (Figure 8C), whereas the MLKL inhibitor GW806742X failed to inhibit CDC in Bid KO MEFs (Figure 8F). The latter findings indicate that MLKL activity in CDC is regulated by Bid but not by JNK.

## Discussion

The necrotic CD induced by the complement is currently poorly characterized. Deposits of C5b-9 complexes have been reported in numerous diseased tissues and C5b-9 has been implicated in several diseases as a causative agent, as an inflammatory trigger, or as a protector from tissue injury (47–49). Insights into the mechanisms triggering inflammation and CDC will pave the way to developing novel therapies for complement-associated diseases. CDC is regarded as a CD type associated with a rapid rise in the concentration of intracellular Ca^2+^ ions (5), leading to mitochondrial damage, metabolic depletion (50), and osmotic lysis. However, based on results showing that CD by complement is not affected by preventing cell swelling, it was concluded that the CDC of nucleated cells is not colloid osmotically mediated (51, 52). In addition, the chelation of extracellular calcium ions was shown to delay but not block metabolic depletion and CD (50). In support of this, calcium ion chelation only partially reduces the C5b-9-induced CD of B-leukemia cells (53, 54). Hence, it may be suggested that the complement-induced rise in intracellular calcium ions essentially acts as an initiator and/or enhancer of the CD process. JNK and Bid are new players involved in CDC (10, 11). The results presented here indicate for the first time that RIPK1, RIPK3, and MLKL are pro-death regulators in complement-mediated CD in human and mouse cells. These are known key regulators of necroptosis induced by TNFα, Fas ligand, TRAIL, LPS, and many other inducers (17, 18, 20). A substantial desensitization of cells to CDC occurs after inhibition of RIPK1, RIPK3, or MLKL by Nec-1, GSK’872, or NSA, respectively, and after the specific silencing of these proteins. Accordingly, RIPK3- or MLKL-knockout mouse fibroblasts are more resistant to CDC than are WT fibroblasts. In line with this, overexpression of either RIPK1 or RIPK3 enhances cell susceptibility to complement-induced necrosis.

The kinase function of RIPK1 and RIPK3 is essential for TNFα-induced necroptosis. Nec-1 inhibits the kinase activity of RIPK1 and thus disables RIPK1/RIPK3 interaction and blocks TNFα-induced necroptosis (12, 27). In addition, a kinase-dead RIPK3 mutant cannot signal in TNFα-induced necroptosis (27, 29). Nec-1 was shown here to be protective from C5b-9-induced necrosis in leukemia (K562), colon adenocarcinoma (HT-29), and breast cancer (BT474) cells. Moreover, as shown here, K50A RIPK3 kinase-dead mutant fails to enhance the sensitivity of K562 cells to C5b-9. Thus, like the necroptotic signal induced by TNFα, complement also activates RIPK1 and RIPK3 kinase activity to induce necrotic CD. The fact that Nec-1 treatment has no effect on the CDC of mouse fibroblasts lacking RIPK3 supports the notion that RIPK1 and RIPK3 act together to activate CDC.

TNFα-induced necrosome formation in which RIPK1 and RIPK3 interact through their RHIM domains occurs 2–6 h after TNFR1 activation (19, 27, 29). The effect of complement on the RIP kinases is rapid. RIPK1–RIPK3 binding, indicative of necrosome formation, occurs within 5 min of complement activation. Apparently, RIPK1–RIPK3 interaction through their RHIM domains is also essential for CDC. Unlike native RIPK1, the overexpression of a RIPK1 RHIM ALAA mutant in K562 cells had no effect on their sensitivity to CDC.

The fact that NSA and GW806742X were able to partially protect K562 cells and mouse fibroblasts, respectively, from C5b-9-induced death suggests that MLKL is a potential target of RIPK3 in the complement-induced necrosis pathway. In support of this, evidence is presented that MLKL KO mouse cells or MLKL siRNA-silenced K562 cells are markedly more resistant to C5b-9 than are WT cells. The mode of action of MLKL as a mediator of necrotic CD downstream of TNFR1-activated RIPK3 has been extensively investigated. As reported, phosphorylated RIPK3 recruits and phosphorylates MLKL, thus inducing a conformational change in MLKL (55–57) that facilitates its translocation to the plasma membrane as well as trimerization, by recruiting Ca^2+^ or Na^+^ ion channels and/or membrane permeabilization leading to cell rupture (36, 42–44, 58). Thus, activated MLKL and C5b-9 share their capacity to sever membranes. As shown here, after a short exposure to complement, MLKL relocates to the plasma membrane and is extensively co-localized with C5b-9. Besides confocal microscopy analysis, the claim that MLKL is associated with C9 and C5b-9 is further supported by co-IP analysis. We propose that by activating RIPK3, complement induces relocation of MLKL to the plasma membrane and together C5b-9 and MLKL induce a more potent necrotic CD. The shown binding of membrane-inserted C5b-9 to RIPK3 may help attract MLKL to the vicinity of the plasma membrane, thus facilitating its membrane insertion. The inability of the RIPK3 inhibitor GSK’872 to affect CDC in MLKL-deficient cells supports such RIPK3–MLKL cooperation during complement attacks. However, apparently MLKL’s pro-necrotic effect during CDC is RIPK1 independent because Nec-1’s effect on CDC was similar in WT and MLKL-deficient cells. It is hypothesized that complement activates three cell signaling pathways: (a) RIPK1-, PIPK3-, and MLKL-independent, Ca^2+^-dependent, (b) RIPK1- and RIPK3-dependent, but MLKL independent, and (c) RIPK3- and MLKL-dependent but RIPK1 independent. These pathways may reinforce each other or be mutually exclusive.

Necrostatin-1 has no effect on CDC in JNK1 or Bid knockout fibroblasts. This place Bid and JNK1 (not JNK2) in the RIPK1-mediated signaling pathway of CDC. Indeed, we have shown that unlike C5b-9-mediated death of WT cells, cells lacking Bid are not affected by the JNK inhibitor SP600125 (10). The fact that the inhibitory effects of SP600125 on RIPK3 KO cells and those of GSK’872 on Bid KO cells were much less pronounced as compared with their effect on WT cells places JNK and Bid in the C5b-9-induced RIPK3-dependent necrotic pathway. Various RIP kinase activators, including TNFα and DNA damage, have been shown to activate JNK or Bid cleavage *via* RIPK1 or RIPK3 (59–63). Apparently, TNFα-induced necroptosis can involve Bid (64). Thus, our results are in agreement with earlier data and suggest that JNK and Bid are involved in RIPK-dependent, C5b-9-mediated necrotic CD. Since GW806742X had no effect on the CDC of Bid KO cells, whereas SP600125 efficiently inhibited the CDC of MLKL KO cells, it is conceivable that Bid signals CDC by two distinct pathways: one dependent on RIPK3 and MLKL and one dependent on RIPK1, RIPK3, and JNK.

Confocal fluorescence microscopy imaging of C5b-9, RIPK1, RIPK3, and MLKL in cells exposed to sublytic complement displayed co-localizations between these molecules. This suggests that direct or indirect molecular interactions exist between C5b-9 and RIPK3 as well as between C5b-9 and MLKL in the vicinity of the plasma membrane, and that RIPK1 interacts with RIPK3 throughout the cytoplasm. This is further supported by data showing that direct interactions exist between C5b-9 and MLKL as well as between RIPK1 and RIPK3. These interactions occur a few minutes after the cell membrane deposition of C5b-9 complexes and supposedly amplify the CD event. Thus, upon complement activation, death-promoting complexes are formed in the affected cells. The similarities and differences between these complement-induced protein complexes and the TNFα-induced necrosome remain to be investigated. An advanced event involved in the interaction of C5b-9 with the cells is its endocytosis in a caveolin-dependent process and its accumulation in several endocytic compartments, including the endocytotic recycling compartment ERC (46). Twenty or 30 min after C5b-9 deposition, respectively, C5b-9 is co-localized with RIPK1 or MLKL, but scarcely with RIPK3, in the ERC. Whether RIPK1 plays a role in C5b-9 endocytosis or is just carried along with it remains to be determined.

Since RIPK1, RIPK3, and MLKL are widely expressed proteins that potentially can cause toxicity, their role in disease has been extensively studied. Indeed, they were shown to be directly involved in many pathological conditions and diseases associated with inflammation and tissue damage (20, 65, 66). For example, high levels of MLKL and RIP3 are associated with inflammatory bowel disease in children (67). Inhibition of RIPK3 or RIPK1 played a protective role from conditions such as atherosclerosis (68), renal ischemia reperfusion (69, 70), and renal graft rejection (69). Since complement is also known to cause pathology in many conditions and diseases (71), it is now tempting to propose that, at least to some extent, the pathophysiological effects of complement are mediated through RIP kinases and MLKL activation. However, this remains to be investigated. Programmed necrosis was also described in erythrocytes treated with bacterial pore-forming toxins specific to human CD59 (72). Programmed necrosis proceeded in the eythrocytes, similar to nucleated cell necroptosis, through RIPK1 and MLKL. Further examination of the role of RIPK1 and MLKL under hemolytic conditions caused by complement is also warranted.

Receptor-interacting protein kinase 3 and MLKL act as tumor suppressors, and thus are valuable therapeutic tools, since many cancers develop necroptosis resistance [reviewed in Ref. (73)]. For example, RIPK3 expression is significantly reduced in leukemia cells of AML patients (74) and in breast cancer tumors (75). Primary chronic B-cell leukemia cells also express low RIPK3 levels and are insensitive to necroptosis (76). SuDHL10 B-cell lymphoma cells have low or undetected levels of RIPK3 and MLKL (77). When considering the efficacy of antitumor therapy based on complement-activating antibodies, it is reasonable to assume that the ability of complement to utilize the RIPKs and MLKL for killing or inhibiting cancer cells will be restricted to those cells that express RIPK1, RIPK3, and MLKL at sufficient levels. Therefore, each primary tumor’s sensitivity to complement-dependent, RIPKs/MLKL-mediated cytotoxicity will have to be assessed individually. Nevertheless, as shown here, complement can still activate CD, albeit to a lower extent, in cells lacking RIP kinases or MLKL. This probably occurs through direct plasma membrane perturbations, elevation of the intracellular calcium to toxic levels, and/or through an additional unknown signaling mechanism. Hence, it is reasonable to assume that antibody-based antitumor therapy may be still effective in necroptosis-resistant cancers.

In summary, our data indicate that complement can activate several necrotic CD pathways, including a necroptotic-like signaling pathway involving RIPK1, RIPK3, and MLKL. This shows remarkable similarities between complement-induced necrosis and necroptosis induced by TNFα, FasL, TRAIL, LPS, and other inducers. Cross talks between C5b-9 and the various ligands activating RIPK1, RIPK3, and MLKL may, therefore, occur. In agreement, TNFα-mediated CD of carcinoma cells was reduced after a brief pretreatment with a sulytic dose of complement (78). Similarly, a brief pretreatment with TNFα was shown to protect K562 cells from C5b-9-mediated death (79). In general, it is likely that at sites of autoimmune reactivity and inflammation involving complement activation, the relative amount and timing of death ligands binding and C5b-9 deposition determine which signaling pathway will dominate and to what extent. However, these interactions are complex and involve many components and may either amplify or inhibit each other.

## Ethics Statement

Ethical approval was given for use of human serum by the local Ethics Committee of Tel Aviv University (reference 20130605_11204919). Approval was given for antibody preparation in rabbits by the local Animal Ethics Committee (reference M-11-064). The rabbits were kept at the animal facilities of Tel Aviv University, which is supervised by two trained and certified Veterinarians. The Animal House adheres to NRC Guidelines “Guide for the Care and Use of Laboratory Animals.”

## Author Contributions

ZF designed the study. ML and ZF wrote the article. ML and ND performed the experiments. NM performed the confocal microscope analyses. All authors approved the manuscript.

## Conflict of Interest Statement

The authors declare that the research was conducted in the absence of any commercial or financial relationships that could be construed as a potential conflict of interest.

## Abbreviations

C5b-9, complement terminal complex composed of C5b, C6, C7, C8, and C9; C9D, C9-depleted serum; CDC, complement-dependent cytotoxicity; KO, knockout; HIS, heat-inactivated serum; NHS, normal human serum; MLKL, mixed lineage kinase domain-like protein; Nec-1, necrostatin-1; Nec-1s, necrostatin-1s, 7-Cl-O-Nec-1; NSA, necrosulfonamide; RIPK1, receptor-interacting protein kinase 1; RIPK3, receptor-interacting protein kinase 3.
