# Supplementary material for: Receptor-Interacting Protein Kinases 1 and 3, and Mixed Lineage Kinase Domain-Like Protein Are Activated by Sublytic Complement and Participate in Complement-Dependent Cytotoxicity
Source: Front Immunol. 2018 Feb 23;9:306. doi: 10.3389/fimmu.2018.00306 (PMC5829068; doi:10.3389/fimmu.2018.00306)
Supplement: Supplementary file 1 [file presentation_1.pdf]

RIPK1, RIPK3 and MLKL are activated by sublytic complement and participate in complement-dependent cytotoxicity

Michal Lusthaus, Niv Mazkereth, Natalie Donin and Zvi Fishelson

Department of Cell and Developmental Biology, Sackler School of Medicine, Tel Aviv University, Tel Aviv 69978, Israel

Supplementary figures

Figure S1

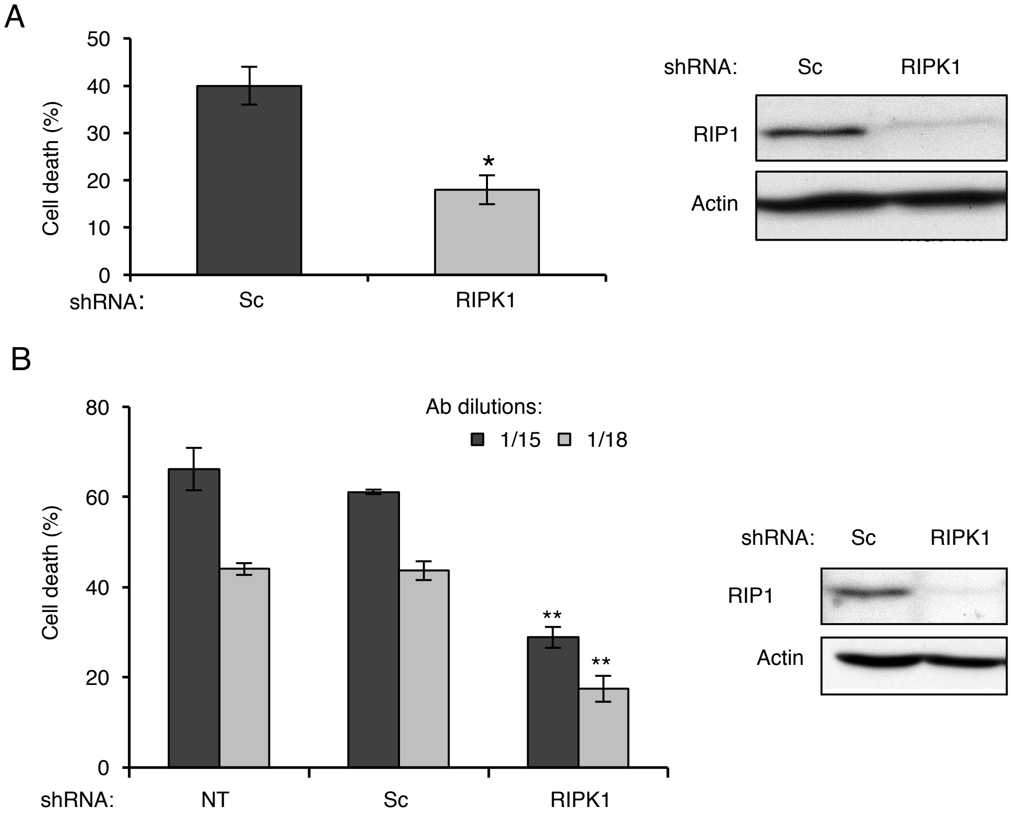

Figure S1. Silencing of RIPK1 by shRNA reduces CDC.

(A) HEK-293T cells transfected for 48 h with RIPK1-shRNA or a scrambled (Sc) shRNA or **(B)** K562 cells stably transfected with RIPK1 shRNA or scrambled shRNA were subjected to antibody and complement (NHS, 50%) treatment for 60 min at 37°C and cell death was measured by PI inclusion. Results of 3 independent experiments are expressed as mean percent cell death ± SD. **P*< 0.05, ***P*< 0.001 relative to cells expressing scrambled shRNA. Knockdown efficiency of RIPK1 was examined by Western blotting of cell lysates with anti-RIPK1 or anti-actin antibodies (right inserts). NT, non-treated.

Figure S2

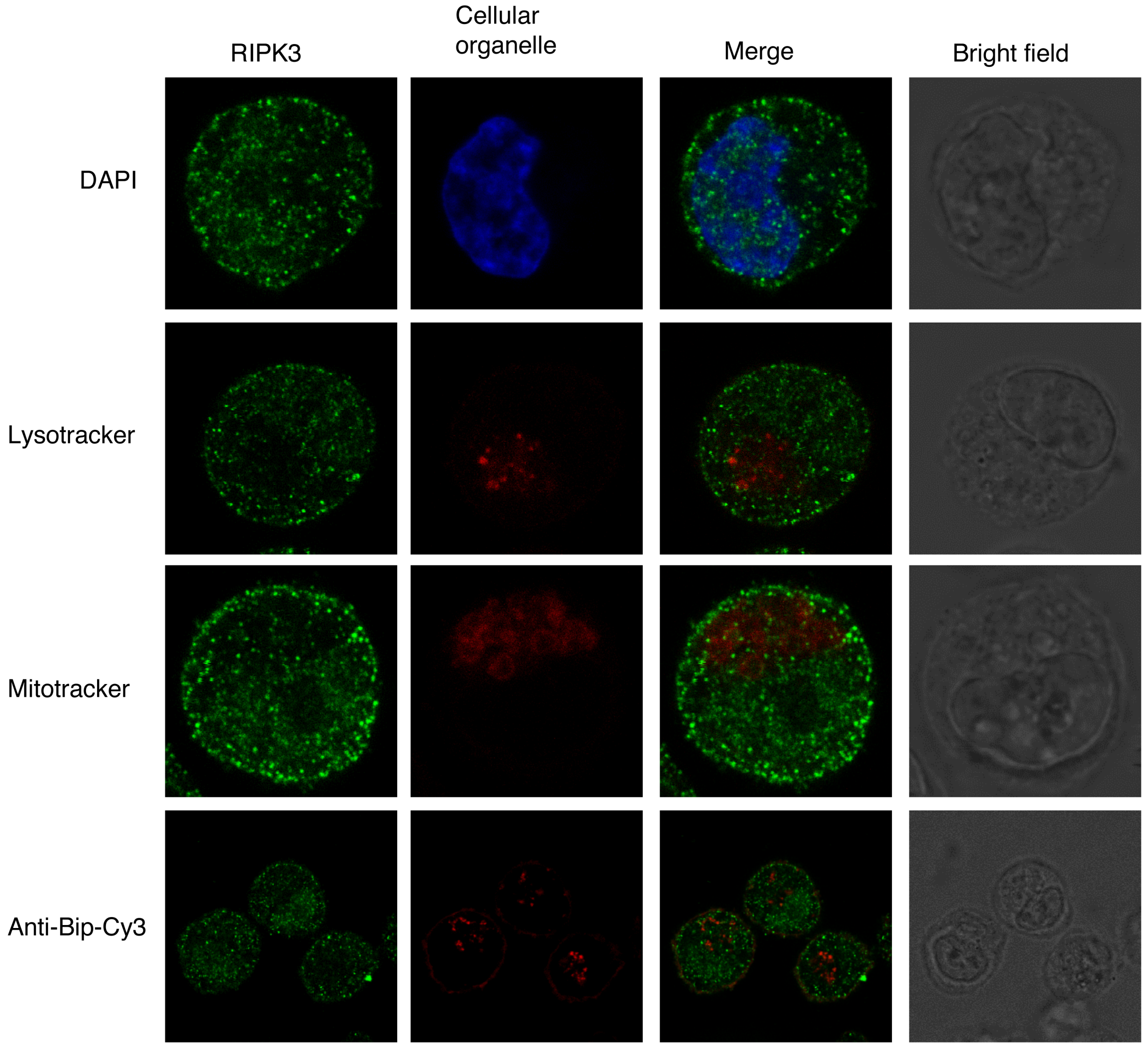

Figure S2. RIPK3 aggregates are not located within cellular organelles.

K562 Flag-RIPK3 transfected cells were treated for 10 min with complement at a sublytic dose, followed by fixation with 2% paraformaldehyde and permeabilization with 0.1% saponin. Flag-RIPK3 was immunostained with AF-488-labeled anti-Flag antibody. Nuclei, lysosomes and mitochondria were stained with DAPI, LysoTracker and MitoTracker, respectively. ER was stained with anti-GRP78 Bip antibody, followed by Cy3 labeled secondary antibody.
